# Murine macrophage TLR2-FcγR synergy via FcγR licensing of IL-6 cytokine mRNA ribosome binding and translation

**DOI:** 10.1371/journal.pone.0200764

**Published:** 2018-07-19

**Authors:** Danielle Hunt, Lisa A. Drake, James R. Drake

**Affiliations:** Albany Medical College, Department of Immunology and Microbial Disease, Albany, NY, United States of America; Chang Gung University, TAIWAN

## Abstract

Macrophages (MØs) are sentinels of the immune system that use pattern recognition receptors such as Toll-like receptors (TLR) to detect invading pathogens and immune receptors such as FcγR to sense the host’s immune state. Crosstalk between these two signaling pathways allows the MØ to tailor the cell’s overall response to prevailing conditions. However, the molecular mechanisms underlying TLR-FcγR crosstalk are only partially understood. Therefore, we employed an immunologically-relevant MØ stimulus, an inactivated gram-negative bacterium that bears TLR2 agonists but *no* TLR4 agonist (iB^TLR2^) opsonized with a monoclonal antibody (mAb-iB^TLR2^), as a tool to study FcγR regulation of TLR2-driven production of IL-6, a key inflammatory cytokine. We chose this particular agonist as an investigational tool because MØ production of any detectable IL-6 in response to mAb-iB^TLR2^ requires *both* TLR2 *and* FcγR signaling, making it an excellent system for the study of receptor synergy. Using genetic, pharmacological and immunological approaches, we demonstrate that the murine MØ IL-6 response to mAb-iB^TLR2^ requires activation of *both* the TLR/NF-κB *and* FcγR/ITAM signaling pathways. mAb-iB^TLR2^ engagement of TLR2 drives NF-κB activation and up-regulation of IL-6 mRNA but fails to result in IL-6 cytokine production/release. Here, Src family kinase-driven FcγR ITAM signaling is necessary to enable IL-6 mRNA incorporation into polysomes and translation. These results reveal a novel mechanism by which FcγR ITAM signaling synergizes with TLR signaling, by “licensing” cytokine mRNA ribosome binding/translation to drive a strong murine MØ cytokine response.

## Introduction

Macrophage (MØ) pattern recognition receptors such as the Toll-like receptors (TLRs) are capable of driving production of key inflammation-associated cytokines such as IL-6. However, it has recently become clear that while Fcγ receptors (FcγRs) can under some conditions induce cytokine production (e.g., [1]), they regularly function to modulate or enhance TLR-driven cytokine production [2]. Yet, the cellular and molecular mechanisms of FcγR enhancement/modulation of TLR-driven cytokine production are only understood at a cursory level. Here, we use an immunologically-relevant MØ stimulant that engages both TLR2 and FcγR to further investigate the cellular mechanism of TLR-FcγR synergy.

For the analysis of receptor crosstalk or synergy, it is most appropriate to use an experimental agonist that possesses a pair of receptor ligands that each on their own drive a sub-maximal response. Under such conditions, receptor crosstalk becomes the main driving factor behind induction of a readily detectable immune response. As detailed below, an inactivated form of the gram-negative bacteria *Francisella tularensis* LVS, which has been opsonized with an IgG anti-LPS mAb is such an agonist and is thus an excellent choice as a tool to study TLR-FcγR crosstalk.

While *F*. *tularensis* LPS is known to *lack* the ability to bind TLR4 [3, 4], Francisella lipoproteins such as Tul4 effectively engage TLR2 and elicit a modest but measurable level of TLR2 activation [5]. Moreover, exposure of MØs to *un*opsonized inactivated Francisella bacteria (i.e., iB^TLR2^) only drives a weak cytokine response [6–8] and vaccination of mice with unopsonized inactivated Francisella fails to generate protective immunity [6, 9–13]. Hence, past analysis suggests that inactivated Francisella alone drives sub-maximal TLR2 signaling. To invoke concurrent FcγR/TLR signaling, inactivated Francisella particles can be opsonized with a murine IgG anti-LPS mAb. The resulting mAb-iB^TLR2^ particles are a good model ligand from a technical perspective because the anti-LPS antibody will *not* bind to (and will thus not occlude) the TLR2 ligands such as Tul4 on the surface of the inactivated bacterium. Moreover, unlike unopsonized iB^TLR2^, mAb-iB^TLR2^ elicits a *robust* MØ cytokine response [6–8]. From a practical perspective, mAb-iB^TLR2^ (aka, mAb-iFt) is a good model as it is an effective Francisella vaccine that protects mice from subsequent challenge with a highly virulent form of the organisms [6, 9–13]. Therefore, we employed mAb-iB^TLR2^ as a model MØ agonist to investigate the mechanism of FcγR enhancement of TLR2-driven cytokine production.

Many different cytokines can elicit an inflammatory response and help drive development of an immune response. Here, we chose to focus on IL-6 because of its central role in many immune responses [14]. In addition to driving development of activated B cells into antibody producing cells, IL-6 can drive Th17 differentiation of naïve helper T cells, development of follicular helper T cells and differentiation of CD8 T cells into cytotoxic cells. However, IL-6 can also have detrimental effects with over-production leading to chronic inflammation and autoimmunity. Hence, IL-6 production needs to be tightly controlled and is thus likely under control of multiple cellular regulatory mechanism (discussed below). Moreover, IL-6 is directly relevant to anti-Francisella immunity as IL-6 knockout mice exhibit a heightened sensitivity to Francisella infection [15].

From a conceptual viewpoint, there are three broad levels at which TLR-FcγR crosstalk or synergy in cytokine production could occur, gene transcription, mRNA translation and/or a post-translational step such as inflammasome activation/pro-cytokine processing or cytokine secretion. At the gene transcription level, convergent TLR and FcγR driven activation of common downstream signaling molecules such as MAPK could synergize to trigger greater signaling pathway activation, resulting in increased cytokine mRNA transcription [16]. At the other end of the process, FcγR signaling could alter inflammasome activation/pro-cytokine processing or the release of non-inflammasome-dependent cytokines. As an example of this mechanism, we have used mAb-iB^TLR2^ (a.k.a. mAb-iFt) to demonstrate that in MØs, FcγR signaling up-regulates activation of the Capsase-1/NLRP3 inflammasome to support enhanced proteolytic processing of TLR2 induced pro-IL-1β to mature IL-1β [7]. In this current report, we investigate the intermediate step of mRNA translation and use mAb-iB^TLR2^ to uncover a novel mechanism underlying FcγR enhancement of TLR2-driven IL-6 cytokine production, FcγR “licensing” of the ribosome binding to and translation of TLR induced IL-6 cytokine mRNA.

## Materials and methods

### Animals

Female C57BL/6J and B6.129-*Tlr2*^*tm1Kir*^/J mice were purchased from Jackson Laboratories. Mice were house and used in strict accordance to the guidelines established by the Albany Medical College Institutional Animal Care and Use Committee. Animal protocols were reviewed and approved by the Albany Medical College Institutional Animal Care and Use Committee (Protocol # 15–03001). Bones from FcRγΔ [17] and FcγIIBΔ (from Taconic) [18] were a gift from Dr. Michelle Lennartz (Albany Medical College).

### Reagents

Mouse anti-TLR2 antibody T2.5 (abcam, ab16894), 1 μg/ml Pam3CSK4 (Tocris, 46331, 1 mg/ml stock in water), 10 μM PP2 (EMD Millipore, 529573, 10 mM stock in DMSO), SuperSignal West Dura chemiluminescent substrate for western blots (ThermoFisher, 34075). Western blots were imaged with a BioRad ChemiDoc Touch Imaging System.

### Generation of bone marrow derived MØ

BMMØ were generated as previously described [19]. Briefly, femur and tibia bone marrow was flushed using a 25G needle attached to a 10 ml syringe filled with cell culture medium. Red blood cells were lysed and the cell suspension was washed prior to final resuspension in BMMØ differentiation media containing 10 ng/ml recombinant m-CSF (Peprotech Cat. # 315–02). Cells were plated in *non-treated* 15 cm petri dishes and placed in a 37°C, 5% CO2 incubator. Cells were supplemented on day 3 with 10 ml additional BMDM differentiation media and harvested using 5 mM EDTA in Ca/Mg-free HBSS on day 7–8.

### Preparation and analysis of mAb-iB^TLR2^

A colony of *F*. *tularensis* Live Vaccine Strain was picked from a chocolate agar plate and expanded in Mueller Hinton Broth to a concentration not exceeding 1x10^9^ organisms/ml. Bacteria were pelleted and washed three times in sterile PBS followed by 2 hours of fixation with 2% Methanol-free Formalin at room temperature. Fixed organisms (i.e., inactivated bacteria, iB^TLR2^) were washed three times in PBS and the concentration measured by absorbance at 610 nm using a standard curve. Aliquot samples were stored at -80°C. Inactivation was confirmed by plating iB^TLR2^ onto chocolate agar for 7–10 days at 37°C and observing no growth. iB^TLR2^ were thawed and resuspended in 1 ml of BMMØ culture media at a concentration of 1x10^9^ iB^TLR2^/ml. Mouse IgG_2a_ mAb against *F*. *tularensis* LPS (clone M0232621, Fitzgerald Industries International cat. # 10-F02B) was added to a final concentration of 5 ug/ml. The mixture was then rotated at 4°C for a minimum of 1 hour prior to use.

### mAb-iB^TLR2^ flow cytometric analysis

GFP-expressing iB^TLR2^ [20], with or without mAb opsonization, were stained with Goat anti-mouse IgG-PE (Southern Biotech 1031–09), washed and analyzed by flow cytometry.

### Differential staining of MØ-associated mAb-iB^TLR2^

Differential straining of extracellular vs. intracellular mAb-iB^TLR2^ was performed as previously reported [21].

### Conditions for BMMØ stimulation with mAb-iB^TLR2^

**Fig 1:**
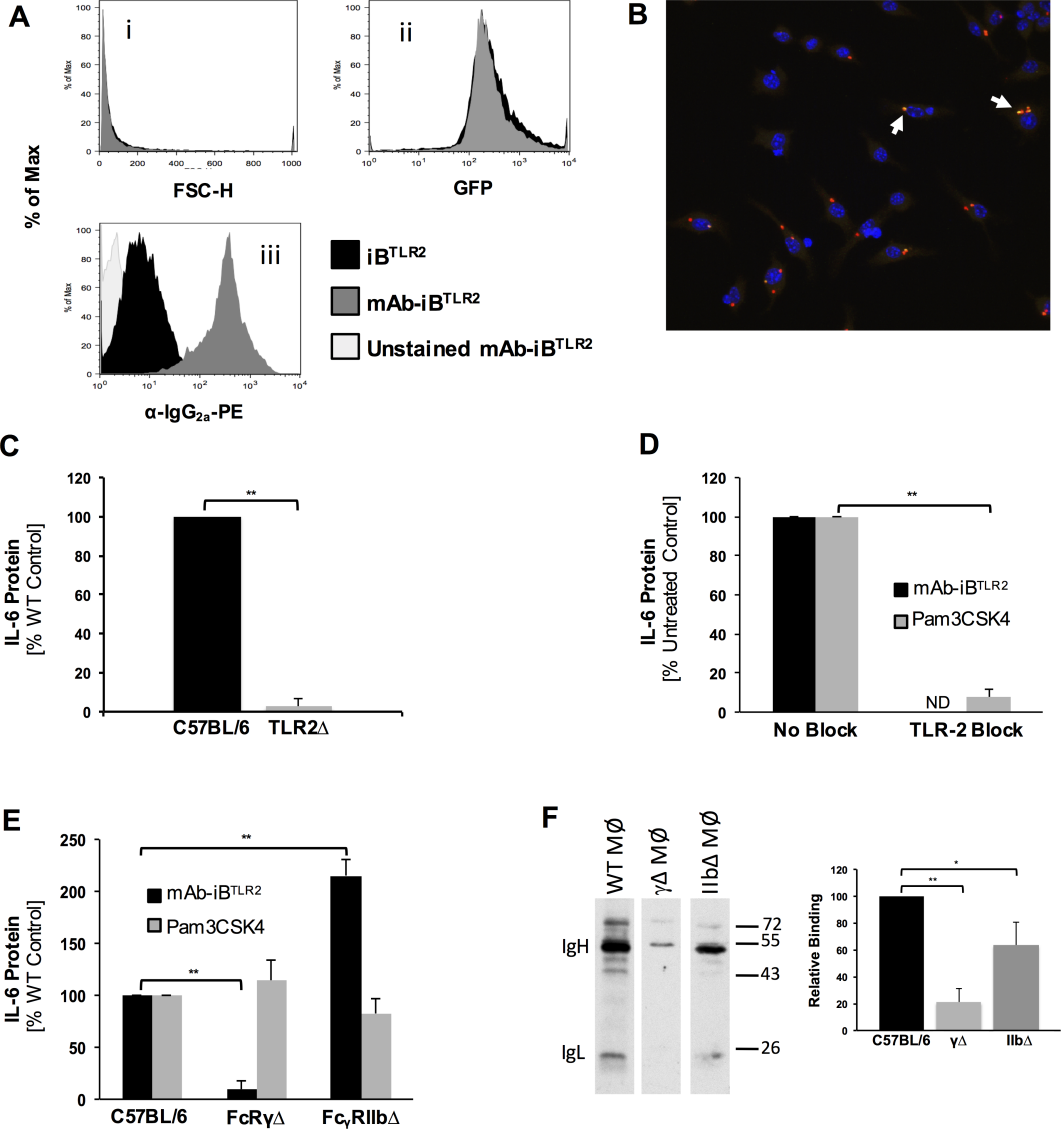
TLR2 and FcγR synergize to drive the Macrophage IL-6 cytokine response to mAb-iB^TLR2^. Panel A: Flow cytometric analysis of mAb-iB^TLR2^. The effect of mAb opsonization on particle size/aggregation was determined by forward light scatter (FCS, sub-panel i) and GFP levels (sub-panel ii). The level of opsonizing IgG mAb binding determined by staining with anti-mouse IgG-PE and analysis by flow cytometry (sub-panel iii, which is gated on GFP-expressing iB^TLR2^). Panel B: BMMØ were pulsed with mAb-iB^TLR2^ for 60 min. at 37°C (see methods), fixed and stained for extracellular mAb- iB^TLR2^ (green/yellow, arrows), intracellular mAb- iB^TLR2^ (red) or DNA (DAPI, blue). Shown is a representative field of view from 1 of 3 independent experiments. Panels C-E: BMMØ were stimulated with mAb-iB^TLR2^ or 1 μg/ml Pam3CSK4 as indicated (TLR2 blocking antibody was used at 10 μg/ml) and the resultant level of culture supernatant IL-6 cytokine determined by ELISA. Shown are IL-6 levels compared to untreated control or control wild type (WT) BMMØ stimulated with mAb-iB^TLR2^, averaged across three or more independent experiments (± 1 S.D). Mean level of IL-6 detected in cultures supernatants from control mAb-iB^TLR2^ stimulated BMMØ was 554 pg/ml (panel C), 3,334 pg/ml (panel D) and 1,147 pg/ml (panel E). Culture supernatants from both unstimulated BMMØ and BMMØ exposed to non-opsonized iB^TLR2^ did not contain detectable levels of IL-6. Panel F: mAb-iB^TLR2^ binding to the indicated BMMØ was determined by western blot analysis of the indicated samples for the opsonizing murine IgG mAb. The signal for the opsonizing mAb heavy chain (IgH) was determined across three independent experiments and is reported relative to WT BMMØ (± 1 S.D.). * = p<0.05, ** = p<0.01.

As previously reported by us [7, 8] and others [22, 23], BMMØ were stimulated with mAb-iB^TLR2^ at an input ratio of 100 mAb-iB^TLR2^ to 1 BMMØ *without centrifugation* as previously reported [8, 22]. We chose *not* to “pellet” the mAb-iB^TLR2^ onto the BMMØ by centrifugation to avoid stressing and possibly activating the MØ. Since the adherent MØ can only sample the contents of the bottom ~10–20 μm of the overlying 1–2 ml of culture media and the mAb-iB^TLR2^ do not readily settle during the incubation, an *applied* 100:1 mAb-iB^TLR2^ to MØ ratio results in an *effective* ratio in the range of 1:1 as evidenced in the IFM staining of MØ for associated mAb-iB^TLR2^ particles (Fig 1B)

### Secreted IL-6 and TNF protein

Following pre-treatment as indicated in figure legends, BMMØ (2x10^6^ in a single well of a 6-well plate per experimental condition) were stimulated with mAb-iB^TLR2^ for 2 hours at 37°C, 5% CO_2_. Cells were then washed and cultured in fresh medium for an additional 18 hours. Cell culture supernatants were analyzed via Mouse IL-6 ELISA Ready-SET-Go! (eBioscience, 88-7064-88]) or Mouse TNF ELISA Ready-SET-Go! (eBioscience, 88-7324-88) according to the manufacturer’s instructions.

### mAb-iB^TLR2^ binding to BMMØ

An excess of mAb-iB^TLR2^ (5 times the standard input) was bound to WT or FcRΔ BMMØ (1x10^5^ MØ in a well of a 96-well plate) for 30 min. *on ice* to prevent non-specific uptake and saturate available receptors. BMMØ were washed to remove unbound mAb-iB^TLR2^, lysed and analyzed for the heavy chain of the opsonizing mAb via SDS-PAGE and western blot with HRP-conjugated goat anti-mouse IgG (Calbiochem, 401253).

### Reactive oxygen species production

mAb-iB^TLR2^ induced BMMØ ROS production was monitored as previously reported [8].

### IκB degradation

Following pre-treatment as indicated in figure legend, BMMØ (1x10^5^ in a single well of a 96-well plate per experimental condition) were stimulated with mAb-iB^TLR2^ at 37°C, 5%CO_2_ over a course of 90 minutes. Cell lysates were prepared at each time point and analyzed via SDS-PAGE and western blot with rabbit anti-IκBα (Cell Signaling Technology, 9242) followed by HRP-conjugated donkey anti-rabbit (BioLegend, 406401). Rabbit anti-β-actin (Cell Signaling Technology, 8457S) was used as a blotting control.

### NF-κB nuclear localization

BMMØ (2.5x10^5^ per experimental condition) were cultured on glass coverslips in a 24-well plate. The following day BMMØ were treated with mAb-iB^TLR2^ at 37°C, 5% CO_2_ for 30 minutes without centrifugation prior to fixation and permeabilization as previously described [8]. Cells were then stained with rabbit anti-mouse NF-κB p65 mAb, clone C22B4 (Cell Signaling Technology, 4764) followed by goat anti-rabbit IgG-Alexa Fluor 594 (Cell Signaling Technology, 8889) and imaged on an Olympus IX81 confocal microscope with Olympus Fluoview FV1000 module, 60X objective (N.A. 1.20 W) using the Olympus Fluoview Ver 2.1c software.

### IL-6 and TNF mRNA

Following pre-treatment as indicated in figure legends, BMMØ (2x10^6^ in a single well of a 6-well plate per experimental condition) were stimulated with mAb-ib^TLR2^ without centrifugation for 2 hours at 37°C, 5%CO_2_. RNA was then isolated using RNeasy mini kit (Qiagen, 74104) and 1 ug of RNA reverse transcribed into cDNA using Transcriptor Reverse Transcriptase (Roche, 03 531 295 001). cDNA was subsequently used for gene expression analysis via quantitative real time PCR (BioRad CFX96 Real-Time System) using SsoAdvanced Universal SYBR Green Supermix (BioRad 1725270) in combination with PrimePCR SYBR Green Assay for Mouse IL-6 (BioRad 10025636, qMmuCID0005613) and PrimePCR SYBR Green Assay for Mouse GAPDH (BioRad 10025636, qMmuCED0027497). The threshold cycle (Ct) values were then used in accordance with the 2^-ΔΔCt^ method of analysis [24], with GAPDH as the reference.

### Intracellular IL-6 protein

BMMØ (2.5x10^5^ per experimental condition) were cultured on glass coverslips in a 24-well plate. The following day cells were incubated for 1 hour at 37°C, 5%CO_2_ with media ±10 μM PP2. Cells were then stimulated with mAb-iB^TLR2^, 1 μg/ml *E*. *coli* LPS, or 1 μg/ml Pam3CSK4 and then cultured for 18 hours. Brefeldin A (Sigma, B7651) was then added to each well to a final concentration of 1 µg/ml and the samples incubated for an additional 3 hours at 37°C. Cells were then washed two times with PBS and fixed as previously described [8]. Samples were stained with rabbit anti-mouse IL-6 mAb D5W4V (Cell Signaling Technology, 12912), followed by goat anti-rabbit IgG- Alexa Fluor 594 (Cell Signaling Technology, 8889) and imaged on an Olympus IX81 confocal microscope (details above).

### Polysome isolation and analysis

BMMØ (1x10^7^ in a 10 cm plate per experimental condition) were incubated for 1 hour at 37°C, 5%CO_2_ with media ±10 μM PP2. Cells were then stimulated with mAb-iB^TLR2^ for 2 hours, washed once with PBS and treated for 10 min. with PBS containing 100 μg/ml cycloheximide (Sigma C1988) to “lock” ribosomes onto the mRNA. Cells were then lysed on ice in 1.0 ml cold polysome lysis buffer [20 mM Tris, pH 8.0, 140 mM NaCl, 15 mM MgCl_2_, 0.5% Triton X-100, 100 μg/ml cycloheximide, 100U/ml RNasin (Promega N2515) and protease inhibitor cocktail (ThermoScientific 88666)]. Lysates were homogenized by passing several times through a 25-gauge needle attached to a 1 ml syringe and then centrifuged for 15 min at 4°C, 13,000 rpm. One ml of cleared lysate was layered onto an 11 ml 10%-50% sucrose gradients and the gradients centrifuged in an SW41 Ti rotor at 35,000 rpm for 2 hours. Twelve 1.0 ml fractions were collected, and each fraction analyzed for refractive index, ribosomal protein and mRNA.

To monitor the presence of ribosomes across the gradient, fractions were analyzed via SDS-PAGE and western blot with rabbit anti-ribosomal protein L26 (abcam, ab59567) followed by HRP-conjugated donkey anti-rabbit (BioLegend, 406401). Control blots were probed with mouse anti-GAPDH (Ambion, AM4300) followed by HRP-conjugated goat anti-mouse (Calbiochem, 401253).

For mRNA analysis, a 250 μl samples of each sucrose gradient fraction were spiked with 1 ng of luciferase RNA (Promega, L4561) to provide both a control for the efficiency of RNA recovery and an exogenous reference for qPCR analysis [25]. RNA was then isolated with TRIzol LS reagent (Life Technologies, 10296010) and treated with DNAse I (Roche, 04716728001). mRNA levels were measured via quantitative real time PCR (BioRad CFX96 Real-Time System) using iTaq Universal SYBR Green One-Step Kit (BioRad, 1725150) in combination with PrimePCR SYBR Green Assay for Mouse IL-6 (BioRad, 10025636, qMmuCID0005613), PrimePCR SYBR Green Assay for Mouse GAPDH (BioRad, 10025636, qMmuCED0027497) or luciferase primer pair (luc-F ACGTCTTCCCGACGATGA and luc-R GTCTTTCCGTGCTCCAAAAC). For each fraction the threshold cycle (Ct) values for IL-6 and GAPDH were corrected by subtraction of the Ct value of the exogenous luciferase reference. The average corrected Ct value (average ΔCt) across the gradient was determined and the mRNA level of each fraction calculated as a fold difference from the average.

## Results

### The Macrophage IL-6 cytokine response to mAb-iB^TLR2^ requires both TLR2 and FcγR-ITAM signaling

To generate the ligand used throughout these studies, inactivated *F*. *tularensis* LVS bacteria (iB^TLR2^) were opsonized with a murine IgG_2a_ anti-LPS mAb. Previous studies have shown that Francisella grown under different conditions (i.e., MHB vs. BHI media) exhibit differences in bacterial physiology and protein expression [26], and that MHB-grown Francisella binds more mAb (because of decreased O-antigen production) than BHI-grown Francisella [27]. Therefore, we used mAb-iB^TLR2^ made from MHB-grown Francisella for the studies detailed below. The level of mAb opsonization was determined by staining of mAb-iB^TLR2^ with a fluorescently labeled anti-mouse IgG antibody and analysis of the stained bacteria by flow cytometry (Fig 1A). The results demonstrate that opsonization conditions do *not* result in appreciable aggregation of the inactivated Francisella (which would have resulted in an increase in either forward light scattering and/or GFP level per particle/event) and a similar level of mAb binding to all bacteria.

To investigate the roles of TLR2 and FcγR in the MØ response to mAb-iB^TLR2^, bone marrow-derived MØ (BMMØ) from wild type (WT) and gene knockout (Δ) mice were stimulated with mAb-iB^TLR2^ for 2 hours at 37°C, washed to remove non-MØ associated particles and IL-6 protein secretion at 18–20 hours monitored by ELISA. Under these conditions, WT BMMØ produce robust levels of secreted IL-6 cytokine. Moreover, blockade of TLR2 engagement/signaling by either use of TLR2Δ BMMØ (Fig 1C) or addition of an anti-TLR2 blocking antibody (Fig 1D) completely ablates the MØ IL-6 response. This finding establishes a unique and central role for TLR2 in the MØ IL-6 response to mAb-iB^TLR2^ (consistent with the presence of multiple TLR2 ligands on the bacterial surface [5]), and reveals that mAb-iB^TLR2^ cannot effectively engage other MØ TLR such as TLR4 to drive an IL-6 response (consistent with the lack of Francisella TLR4 ligands [4]).

Macrophages express two broad classes of FcγR. One is the activating FcγRs such as CD64 (FcγRI), all of which associate with the FcRγ chain (FcR γ) that has a cytoplasmic immunoreceptor tyrosine-based activation motif (ITAM). Engagement of ITAM-bearing FcγRs drives activation of src-family kinases and production of reactive oxygen species (ROS), both of which are key mediators of FcγR ITAM signaling [8]. The second class of FcR is the inhibitory FcγR (i.e., FcγRIIB), which possess an immunoreceptor tyrosine-based inhibition motif (ITIM). Engagement of ITIM-bearing FcγRs results in recruitment/activation of phosphatases such as SRC-homology-2-domain-containing inositol-5-phosphatase (SHIP) [23], which counters FcγR ITAM signaling. To investigate the role of these two classes of FcγR in the MØ IL-6 response to mAb-iB^TLR2^, BMMØ with a specific deletion of either the ITAM-bearing FcR γ chain (FcRγΔ) or ITIM-bearing inhibitory FcR (FcγRIIBΔ) were stimulated with mAb-iB^TLR2^ and IL-6 protein secretion monitored (Fig 1E). Exposure of FcRγ Δ BMMØ to mAb-iB^TLR2^ fails to elicit substantial IL-6 production even though the TLR2-MyD88 signaling pathway is intact in these cells (Fig 1E, Pam3CSK4 stimulation), suggesting a necessary role for *both* TLR2 *and* FcγR ITAM signaling in the MØ IL-6 response to mAb-iB^TLR2^. In addition, mAb-iB^TLR2^ stimulation of BMMØ lacking the inhibitory FcγRIIB results in an approximately 2-fold increase in the IL-6 response, suggesting that mAb-iB^TLR2^ engagement of the inhibitory FcR partially counteracts FcγR ITAM signaling to blunt the MØ IL-6 response. This last finding is in line with the observation that FcγRIIB can blunt the protective *in vivo* immune response elicited by mAb-iB^TLR2^ vaccination [10]. As a control, we monitored the response of all BMMØ to the synthetic TLR2 ligand Pam3CKS, which is essentially the same across all cell types (Fig 1E, gray bars).

The profoundly decreased IL-6 response of FcRγΔ BMMØ to mAb-iB^TLR2^ could be due either to decreased FcγR signaling *or* decreased mAb-iB^TLR2^ binding due to reduced levels of FcγR expression. To investigate these possibilities, we determined the level of mAb-iB^TLR2^ binding to wild type and FcRγΔ BMMØ (Fig 1F). Here, we measured mAb-iB^TLR2^ binding to BMMØ at reduced temperature so that binding would be a direct readout of FcγR expression. Under these conditions, we observe an approximately 70% decrease in mAb-iB^TLR2^ binding to the FcRγΔ BMMØ. However, this decrease in mAb-iB^TLR2^ binding was less dramatic than the ~90% decrease in IL-6 cytokine production by these MØs (Fig 1E), *suggesting* an additional effect of the absence of ITAM signaling on IL-6 production and prompting us to further investigate the role of FcγR ITAM signaling in the MØ IL-6 response (below). Interestingly, while the level of mAb-iB^TLR2^ binding to FcγRIIBΔ BMMØ was decreased by about 50% (Fig 1F), these cells produce approximately twice as much IL-6 as WT BMMØ (Fig 1E), reinforcing the idea that there is a significant inhibitory effect of engagement of the ITIM-bearing FcγRIIB on the MØ IL-6 response to mAb-iB^TLR2^ (which is alleviated in the FcγIIBΔ BMMØ).

**Fig 2:**
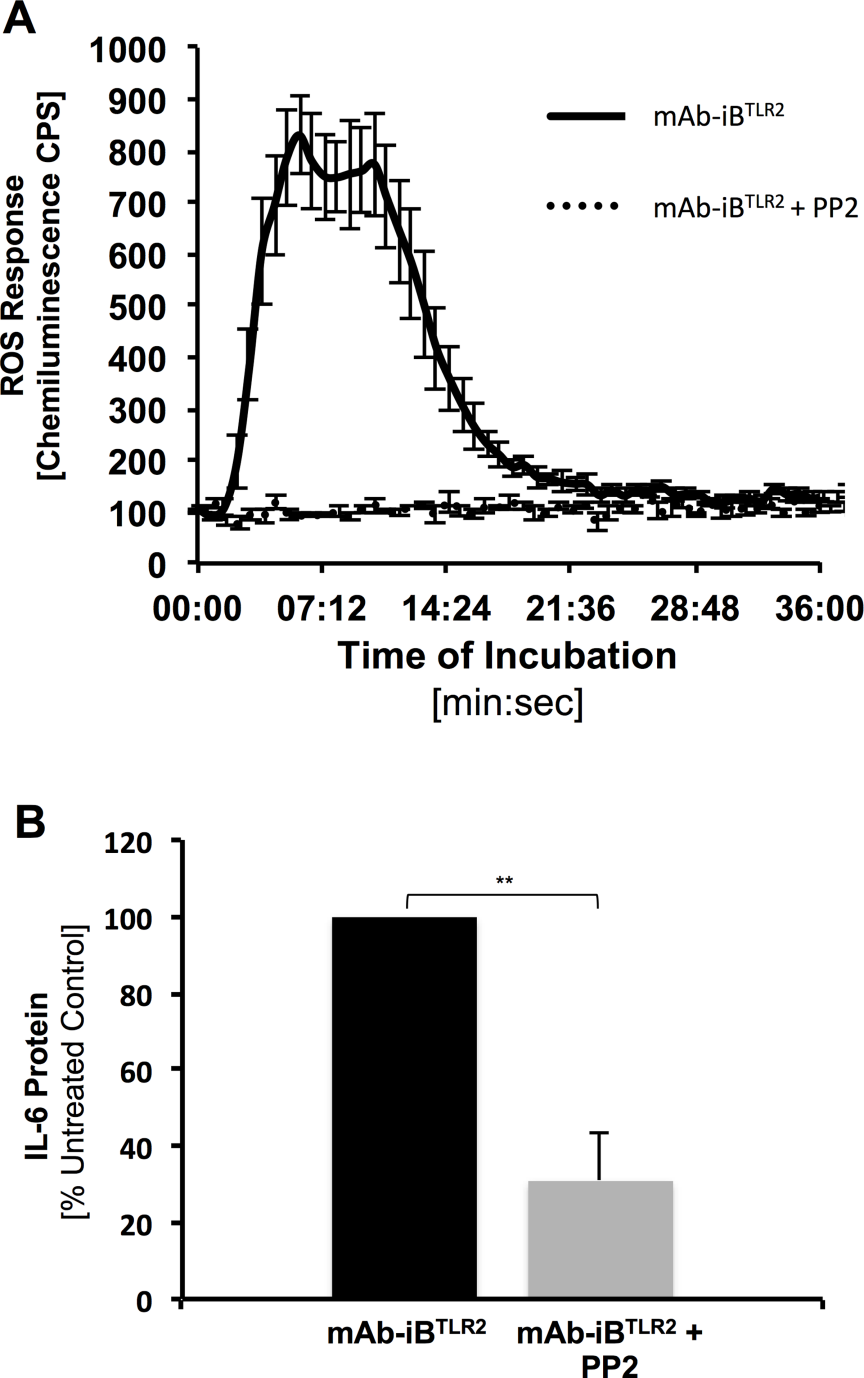
FcγR ITAM signaling is necessary for the Macrophage IL-6 cytokine response to mAb-iB^TLR2^. Panel A: BMMØ were stimulated with mAb-iB^TLR2^ under the indicated conditions and the resultant level of ROS production determined by luminol assay. Shown are results averaged across 6 independent experiments. Error bars indicate ± 1 S.E.M. Panel B: The indicated BMMØ were stimulated with mAb-iB^TLR2^ under the indicated conditions and the resultant level of IL-6 cytokine in the culture supernatant determined by ELISA. The mean level of IL-6 detected in cultures supernatants from control mAb-iB^TLR2^ stimulated BMMØ was 776 pg/ml. Shown are IL-6 levels compared to untreated BMMØ across three independent experiments (± 1 S.D). ** = p<0.01.

To further investigate the potential role of ITAM-bearing FcγRs in the MØ response to mAb-iB^TLR2^, we took a pharmacological approach that would *not* result in a change in MØ FcγR expression and concomitant change in mAb-iB^TLR2^ binding. mAb-iB^TLR2^ engagement of ITAM-bearing FcγR results in activation of Src family kinases and subsequent induction of NOX-dependent ROS production, both key elements of FcγR signaling [8]. Therefore, to pharmacologically block activating FcγR ITAM signaling we employed the Src family kinase inhibitor PP2 [7]. We confirmed that exposure of BMMØ to mAb-iB^TLR2^ results in a robust FcγR/ITAM-driven ROS response [8], which is profoundly inhibited by acute treatment of the MØ with 10 μM PP2 (Fig 2A). Turning to IL-6 production, we can see that pharmacological blockade of FcγR ITAM signaling with PP2 profoundly inhibits the BMMØ IL-6 response to mAb-iB^TLR2^ (Fig 2B), confirming a key role for FcγR-based ITAM signaling in the MØ IL-6 response to this agonist *even in the presence of unfettered TLR2 signaling*.

Taken together, the results presented in Figs 1 and 2 demonstrate a true synergy between TLR2 and FcγR ITAM signaling to drive the MØ IL-6 cytokine response to mAb-iB^TLR2^. If we singly block either TLR2 signaling (Fig 1C and 1D) or FcγR ITAM signaling (Figs 1E and 2) there is a profound blockade in MØ IL-6 cytokine production. Only when *both* signals are present does one obtain robust levels of IL-6 cytokine production.

### TLR2 drives an NF-κB response to mAb-iB^TLR2^ to elicit IL-6 mRNA

Production of immunologically active IL-6 cytokine involves three basic steps; transcription of the IL-6 gene into IL-6 mRNA, translation of IL-6 mRNA into IL-6 protein and release of IL-6 protein from the cell. To determine if TLR2-FcγR synergy is occurring at a pre-transcriptional step in IL-6 cytokine production, we first investigated the effect of FcγR engagement on the activation of TLR2-induced signaling pathways and induction of IL-6 mRNA. NF-κB is a primary driver of cytokine mRNA transcription and TLR2 is well-known to signal via MyD88 to drive NF-κB activation [28]. However, ITAM-based signaling can also lead to NF-κB activation [29]. Thus, mAb-iB^TLR2^ could activate NF-κB by two distinct pathways, possibly resulting in increased IL-6 gene transcription. Therefore, we sought to determine the relative roles of TLR2 and FcγR ITAM signaling in NF-κB activation in response to mAb-iB^TLR2^.

**Fig 3:**
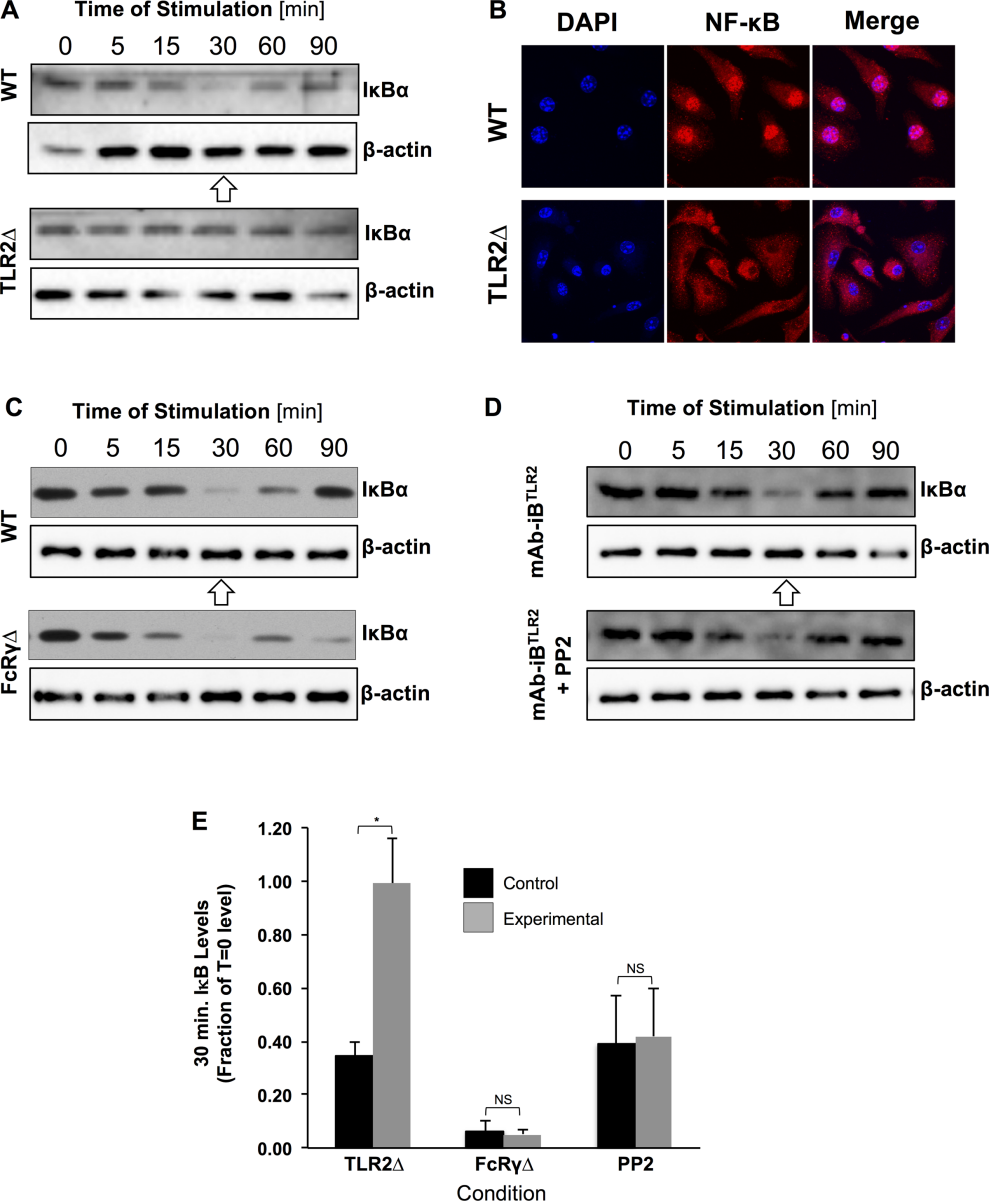
NF-κB activation by mAb-iB^TLR2^ requires TLR2 but not FcγR ITAM signaling. Panel A: WT and TLR2Δ BMMØ were stimulated with mAb-iB^TLR2^ for the indicated time and total cellular IκB levels determined by western blot. Panel B: WT and TLR2Δ BMMØ were stimulated with mAb-iB^TLR2^ for 30 minutes (arrow in Panel A). The cells were then fixed and stained for NF-κB and DNA (DAPI) and then imaged by confocal microscopy. Panels C and D: The indicated BMMØ were stimulated with mAb-iB^TLR2^ and IκB degradation monitored as in panel A. For all panels, shown are representative results from 1 of 3 independent experiments. Panel E: Fraction of initial IκB levels observed 30 min. post stimulation for each sample (error bars = ± 1 S.D.). * = p<0.05. NS = not statistically significant.

While NF-κB activation is a complex multi-step process, two key activation steps are degradation of the inhibitory subunit IκB and nuclear localization of the liberated NF-κB molecule. To investigate the possibility of NF-κB as a node of TLR2-FcγR crosstalk, we started by analyzing mAb-iB^TLR2^ driven NF-κB activation in wild type (WT) and TLR2Δ BMMØ by monitoring IκB levels by western blot (Fig 3A). As anticipated, exposure of WT BMMØ to mAb-iB^TLR2^ results in IκB degradation that is maximal at 30 minutes, followed by a typical recovery of IκB levels by 90 minutes. In stark contrast, exposure of TLR2Δ BMMØ to mAb-iB^TLR2^ fails to drive IκB degradation (Fig 3A and 3E), indicating a central role for TLR2 in mAb-iB^TLR2^ driven NF-κB activation. To determine if the observed TLR2 driven induction of IκB degradation results in NF-κB activation, we investigated the nuclear localization of the p65 subunit of NF-κB in BMMØ 30 minutes after mAb-iB^TLR2^ exposure (Fig 3B), which is the time that corresponds to the greatest degree of IκB degradation (Fig 3A, arrow). The results reveal that stimulation of WT MØ with mAb-iB^TLR2^ results in robust nuclear localization of NF-κB p65 at a time that corresponds with the peak of IκB degradation (i.e., 30 minutes) and that this localization fails to occur in TLR2Δ BMMØ, indicating a central and critical role for TLR2 in the MØ NF-κB response to mAb-iB^TLR2^.

To determine whether FcγR ITAM signaling alters the TLR2 driven NF-κB response to mAb-iB^TLR2^, we analyzed mAb-iB^TLR2^ induced IκB degradation in both FcRγΔ BMMØ (Fig 3C) as well as WT BMMØ treated with PP2 (Fig 3D), which blocks FcγR Src family kinase ITAM signaling (Fig 2). In both cases, the absence/blockade of FcγR ITAM signaling *fails* to result in a detectable change in the pattern or extent of mAb-iB^TLR2^ induced IκB degradation (Fig 3E). Together, the results presented in Fig 3 reveal that TLR2 is critical to activation of the MØ NF-κB signaling pathway in response to mAb-iB^TLR2^ and indicate that FcγR ITAM signaling appears *not* to be acting primarily to modulate this aspect of MØ TLR signaling.

### FcγR ITAM signaling controls IL-6 mRNA ribosome binding and translation

FcγR ITAM signaling is key to the MØ IL-6 cytokine response to mAb-iB^TLR2^ (Figs 1 and 2) but does not appear to primarily function by augmenting or modulating TLR2-driven NF-κB activation (Fig 3), which is key for IL-6 gene transcription. Therefore, we sought to investigate the impact of ITAM signaling on the later steps of IL-6 protein biosynthesis/secretion (i.e., mRNA translation and IL-6 protein secretion). To get a more complete picture of the roles of these two receptors in elicitation of IL-6 production, we determined the roles of both the TLR2 receptor and FcγR ITAM signaling in the production of both IL-6 mRNA and IL-6 protein.

**Fig 4:**
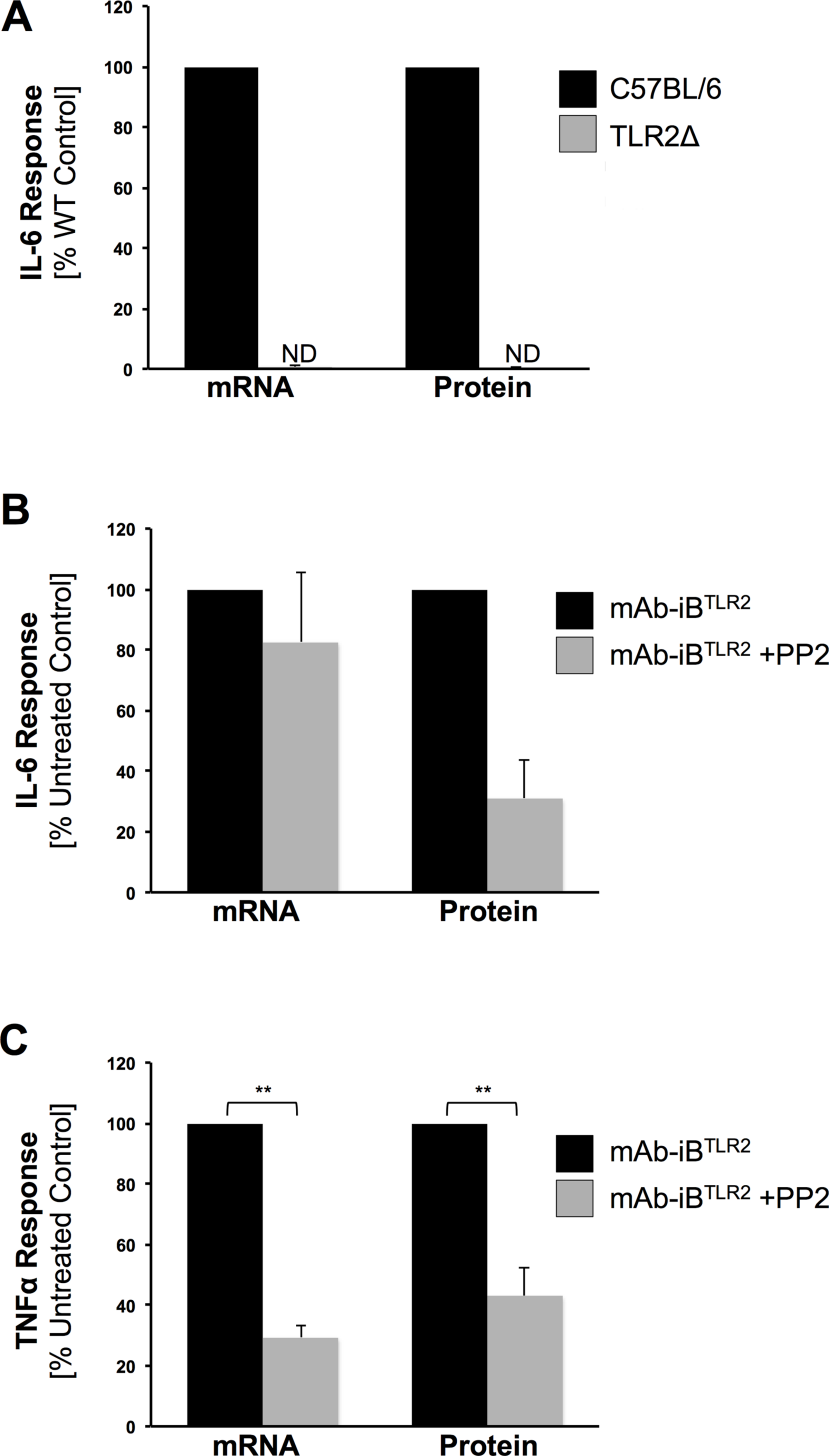
mAb-iB^TLR2^ induction of IL-6 mRNA requires TLR2 but not FcγR ITAM signaling. BMMØ were stimulated with mAb-iB^TLR2^ under the indicated conditions. The level of cellular mRNA was determined by RT-PCR (see materials and methods) and the level of secreted IL-6 or TNF protein determined by ELISA (ND = not detected). The mean level of cytokine detected in cultures supernatants from control mAb-iB^TLR2^ stimulated BMMØ was 554 pg/m of IL-6 (panel A), 776 pg/ml of IL-6 (panel B) and 670 pg/ml of TNF (panel C). Show are the average levels of cytokine mRNA and protein (normalized to control non-PP2 WT BMMØ) across three or more independent experiments (error bars = ± 1 S.D.). ** = p<0.01.

BMMØ were stimulated with mAb-iB^TLR2^ and the levels of IL-6 mRNA and secreted IL-6 protein determined (Fig 4). As established by previous results, mAb-iB^TLR2^ stimulation of BMMØ results in robust production of secreted IL-6 protein measured in the culture supernatant and this is dependent on *both* TLR2 (Fig 4A) *and* FcγR ITAM signaling (Fig 4B). However, the pattern of IL-6 mRNA expression is notably different. As might be expected from the observation that TLR2 is primarily responsible for mAb-iB^TLR2^ driven NF-κB activation (Fig 3), production of IL-6 mRNA is completely dependent on TLR2 signaling as IL-6 mRNA is not detected in mAb-iB^TLR2^ stimulated TLR2Δ BMMØ (Fig 4A). However, blockade of FcγR ITAM signaling by treatment with the Src inhibitor PP2 *fails* to result in a statically significant decrease in IL-6 mRNA levels (Fig 4B). Thus, BMMØ exposed to mAb-iB^TLR2^ under conditions of blocked FcγR ITAM signaling up-regulate expression of IL-6 mRNA but fail to convert that mRNA into *secreted* IL-6 protein. To investigate the generality of this effect, we analyzed the effect of PP2 on the MØ TNF response to mAb-iB^TLR2^ (Fig 4, panel C), which we have previously shown to also be FcγR dependent [7]. Here, blockade of FcγR ITAM signaling blocks production of *both* TNF mRNA *and* protein, suggesting distinct mechanisms by which FcγR signaling regulates the MØ TNF vs. IL-6 cytokine response to mAb-iB^TLR2^. Together, these results reveal that FcγR ITAM signaling is uniquely acting at a post-transcriptional step to control MØ IL-6 protein biosynthesis and/or secretion in response to mAb-iB^TLR2^ stimulation.

**Fig 5:**
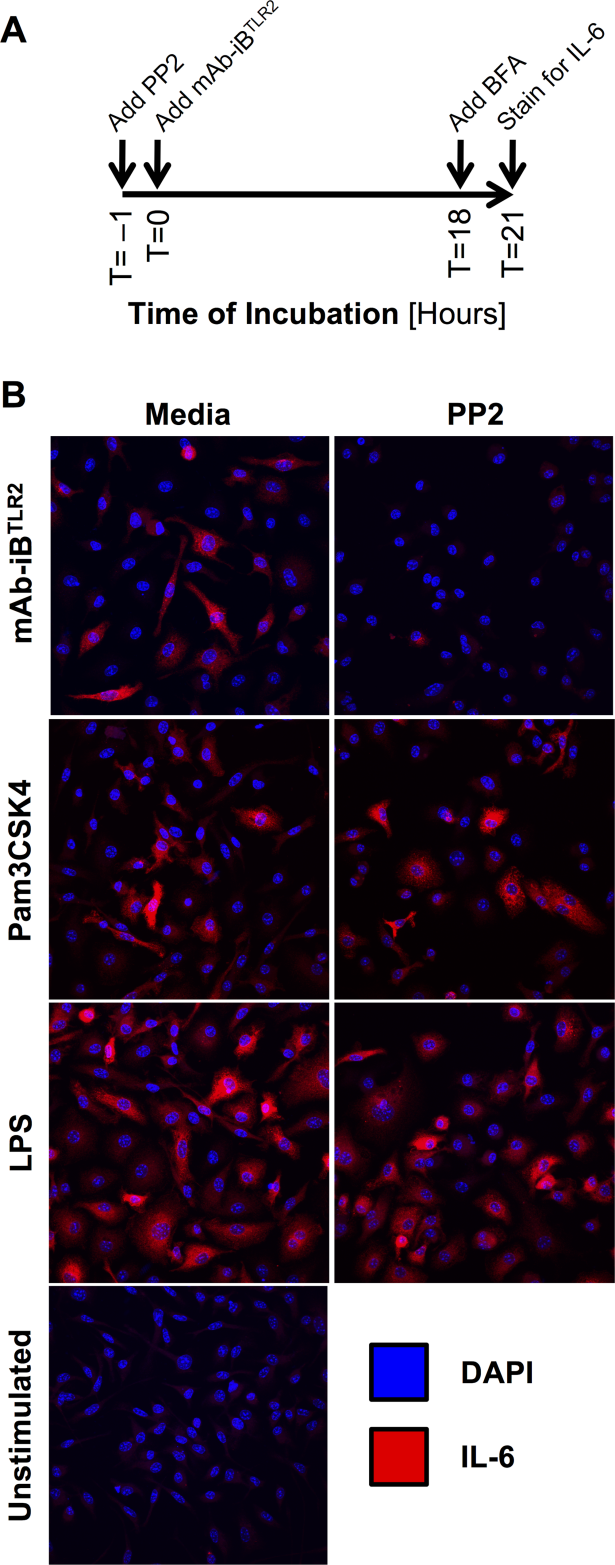
FcγR ITAM signaling facilitates IL-6 mRNA translation. Panel A: Experimental time-line. BFA: Brefeldin A. Panel B: BMMØ stimulated with mAb-iB^TLR2^, Pam3CSK4 or *E*. *coli* LPS were treated with Brefeldin A (to result in intracellular accumulation of any produced IL-6 protein), fixed, permeabilized, stained for IL-6 cytokine and imaged by confocal microscopy. Shown are representative fields from 1 of 3 independent experiments.

There are two major post-transcriptional steps in IL-6 protein biosynthesis/secretion that could be regulated by FcγR ITAM signaling, IL-6 mRNA translation or IL-6 protein secretion. To determine which of these is the target of FcγR mediated regulation, we used immunofluorescence microscopy to look for intracellular IL-6 protein in mAb-iB^TLR2^ stimulated BMMØ that were acutely treated with brefeldin A (BFA, to cause intracellular accumulation of any produced IL-6 cytokine) following the time course shown in Fig 5A. Using this approach, intracellular IL-6 protein is readily detectable in BMMØ stimulated with mAb-iB^TLR2^ but is essentially undetectable in BMMØ where FcγR ITAM signaling was blocked with PP2 (Fig 5B), suggesting that FcγR ITAM signaling is most likely controlling IL-6 mRNA translation to IL-6 protein (see below). As a control, BMMØ were stimulated with the robust TLR4 agonist soluble *E*. *coli* LPS or the robust TLR2 agonist Pam3CSK4 (neither of which engage Fc receptors) in the presence or absence of PP2. Here, there is no significant effect of PP2 treatment on IL-6 protein production (Fig 5B), confirming the selectivity of the PP2 effect on mAb-iB^TLR2^ induced cytokine production. In total, the results presented in Figs 4 and 5 reveal that FcγR ITAM signaling is regulating IL-6 protein production (i.e., mRNA translation) as opposed to IL-6 cytokine secretion.

**Fig 6:**
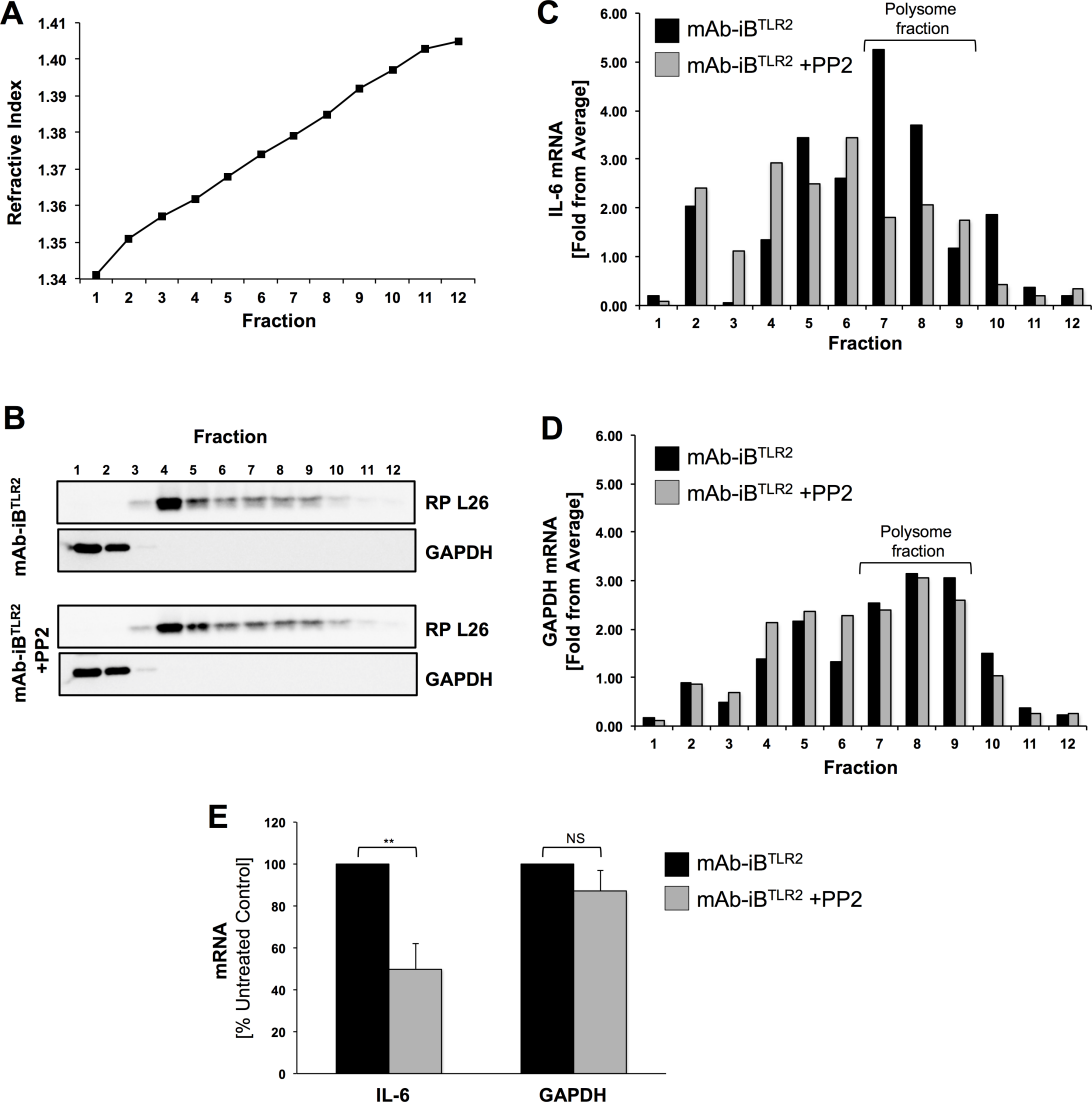
FcγR ITAM signaling drives IL-6 mRNA incorporation into polysomes. BMMØ were stimulated for 2 hours with mAb-iB^TLR2^ in the absence or presence of 10 µM PP2. Cells were then lysed under conditions that stabilize polysomes and the resulting lysates fractionated by sucrose density gradient centrifugation. Panel A: Plot of refractive index to demonstrates the density profile of the sucrose gradient. Panel B: The level of RP L26 (a ribosomal protein) and GAPDH (a soluble cytosolic protein) in each gradient fraction was determined by western blot. Panels C and D: The level of IL-6 and GAPDH mRNA in each gradient fraction was determined by RT-qPCR as detailed in the methods section. Panel E: The average level of mAb-iB^TLR2^-induced IL-6 and GAPDH mRNA (with and without inhibition of FcγR signaling by PP2) in the indicated polysome fractions (panels C and D) across three independent experiments was determined. Error bars = ± 1 S.D. ** = p<0.01.

To gain further insight into how FcγR signaling is controlling IL-6 mRNA translation, we determined the effect of FcγR signaling on IL-6 mRNA incorporation into polysomes, which is a major step in the process of mRNA translation [30]. To accomplish this, WT BMMØ were stimulated with mAb-iB^TLR2^ for 2 hours in the absence or presence of PP2 to block FcγR ITAM signaling. The cells were then lysed under conditions that stabilize polysomes (see methods), and the lysates fractionated by sucrose density gradient centrifugation to separate polysomes from free mRNA and other small molecules. This approach results in the distribution of ribosomes across the density gradient (Fig 6A and 6B), with free ribosomes near the top of the gradient in fractions 3–5 and polysomes closer to the bottom of the gradient in fractions 7–10. We then used an RT-PCR approach to look at the distribution of the IL-6 and GAPDH mRNAs across the gradient (Fig 6C and 6D). GAPDH was included as a control, as the distribution of GAPDH mRNA is *not* expected to be altered by FcγR signaling. Interestingly, blocking FcγR signaling results in a selective decrease in the level of IL-6 mRNA in the polysome-containing region of the gradient (i.e., fractions 7–9), but no change is the distribution of GAPDH mRNA (Fig 6E). This means that FcγR signaling is acting at a step of mRNA translation that controls assembly of IL-6 mRNA containing polysomes, which are platforms for robust cytokine production.

When viewed in total, the results presented in this report reveal a true synergy between TLR2 and FcγR ITAM signaling in the MØ IL-6 cytokine response to mAb-iB^TLR2^. TLR2 engaged by bacterial lipoproteins [5] drives NF-κB activation and IL-6 mRNA production, but not protein production. In contrast, FcγR ITAM signaling works at a post-transcriptional step to drive IL-6 mRNA ribosome binding and translation into IL-6 protein. To our knowledge, this is one of the first time that FcγR ITAM signaling has been shown to regulate MØ inflammatory cytokine production by controlling the efficiency of cytokine mRNA ribosome binding and translation (see below).

## Discussion

The goal of these studies was to gain insight into the cellular and molecular mechanisms which underlie MØ TLR-FcγR synergy/crosstalk. While it is well appreciated that there is functional crosstalk between TLR and FcγR during the induction of MØ pro-inflammatory cytokine production (reviewed in [2, 31]), the cellular and molecular mechanisms underlying TLR-FcγR crosstalk or synergy are poorly understood [31]. Moreover, TLR-FcγR crosstalk occurs in both DCs and MØ and can be either positive or negative (reviewed in [32]) and there can be consequential differences between cells of mouse or human origin (summarized in [2]). In this report, we focus on positive crosstalk as it occurs in murine bone marrow-derived MØ and have identified a novel mechanism by which FcγR signaling facilitates TLR-driven pro-inflammatory cytokine production.

Much of what we know about TLR and FcγR signaling and crosstalk comes from studies using relatively high concentrations of robust receptor agonists, which drives strong receptor signaling. However, this level of receptor engagement and signaling is unlikely to occur under physiological conditions, such as when a MØ interacts with one or a few invading bacteria. Moreover, any analysis of receptor *synergy* would be best carried-out under conditions were sole engagement of either receptor *fails* to elicit a robust response, allowing ready detection of *synergistic* responses. Accordingly, we have investigated TLR-FcγR synergy using mAb-iB^TLR2^ [6, 9–13, 33, 34], which could be considered as a mimic of an antibody-opsonized invading pathogen and which requires engagement of *both* TLR2 *and* FcγR to drive a strong MØ IL-6 response.

In general, FcγR enhancement of TLR-driven cytokine production can occur at either of three broad levels; induction/stabilization of cytokine mRNA, enhanced conversion of cytokine mRNA to cytokine protein or pro-cytokine inflammasome processing/cytokine secretion. The majority of prior studies of TLR-FcγR crosstalk have focused on elicitation of increased cytokine mRNA levels and the potential role of receptor-proximal connections between TLR and FcγR signaling (e.g., MAP kinases) to drive this effect (reviewed in [2, 31]). These studies have implicated FcγR ITAM signaling and the downstream signaling molecules of Src family kinases, Syk, PKC and Raf-1 in enhanced cytokine mRNA production [7, 35, 36]. However, it is unclear how these signaling pathways are interacting to drive enhanced TLR-driven cytokine production [31].

In this report, we focus on MØ production of IL-6 to study TLR2-FcγR synergy. IL-6 is a highly potent pleotropic cytokine that can drive many immune phenomena such as maturation of activated B cells to plasma cells, development of Th17 effector T cells, development of follicular helper T cells and differentiation of CD8 T cells into cytotoxic cells [14]. Moreover, inappropriate IL-6 production can lead to immunopathology. Therefore, it is not surprising that production of IL-6 protein is tightly controlled at multiple levels ([14] and below), providing a rich system in which to investigate the mechanisms of TLR-FcγR synergy.

Induction of IL-6 mRNA is known to be regulated at multiple levels. As recently reviewed [14], TLR signaling results in the activation of transcription factors such as NF-κB, which bind to the IL-6 promotor and drive enhanced mRNA transcription. In addition, TLR signaling also impacts the levels of two proteins that regulate IL-6 mRNA stability. First, TLR signaling *induces* expression of the protein AT-rich interactive domain-containing protein 5A (Arid5a), which binds to the IL-6 mRNA 3’ UTR to *stabilize* the mRNA molecule [37]. Second, TLR signaling drives *degradation* of regulatory RNase-1 (regnase-1, which binds to the 3’ UTR of IL-6 mRNA and mediate its *degradation*), thus increasing IL-6 mRNA stability [38]. Working together, these mechanisms would increase the overall levels of MØ IL-6 mRNA in response to TLR engagement. However, since we do not observe any significant changes (either up or down) in IL-6 mRNA levels upon blockade of FcγR ITAM signaling, it appears that these regulatory mechanisms are *not* involved in FcγR modulation of TLR2-induced IL-6 production.

While less well studied, production of IL-6 cytokine is also controlled at the translational level. In MØ, TLR signaling can result in increased IL-6 mRNA polysome recruitment via a mechanism involving MAP3K mediated dissociation of the inactive 4E-BP–eIF4E complex, liberating the eIF4E translation initiation factor to promote initiation of mRNA translation (see below [39]). In addition, IFN-γ can increase the translation efficiency of IL-6 mRNA in MØs via a mechanism that involves the kinases mTORC1 and MNK [40]. Consistent with the notion that regulation of IL-6 mRNA ribosome binding and initiation of translation is a major control point of IL-6 cytokine production, we report here that FcγR signaling is necessary for the incorporation of TLR2-induced IL-6 mRNA into polysomes and the efficient mRNA translation into IL-6 protein. This represents a significant step forward in our understanding of TLR-FcγR synergy and, as far as we know, the first report of *translational* regulation of MØ gene expression by FcγR ITAM signaling. Of note, a similar mechanism of translational upregulation was recently implicated for TNF production in response to concurrent TLR2/IgA Fc receptor (FcαR) signaling in CD103^+^ human dendritic cells [41] (regulated IL-6 mRNA translation was implicated but not directly not analyzed in this study).

Having determined that FcγR signaling facilitates MØ IL-6 cytokine production by allowing or “licensing” IL-6 mRNA polysome association and subsequent translation, the next step in this study will be the investigation of the underlying molecular mechanism. In CD301^+^ DC, induction of TNF mRNA translation in response to joint TLR2-FcαR engagement is mediated by ITAM-driven glycolytic reprogramming [41], which is known to activate the mTOR– 4E-BP axis (see above). To this end, we have investigated the possible role of 4E-BP in FcγR regulation of MØ IL-6 production using BMMØ from 4E-BPΔ mice (a gift from Dr. Nahum Sonenberg, Table 1). If FcγR signaling was working by driving dissociation of inhibitory 4E-BP from eIF4E to allow eIF4E-catalyzed initiation of IL-6 mRNA translation, blockade of FcγR signaling should *not* have any effect of mAb-iB^TLR2^-induced IL-6 production in 4E-BPΔ MØ (i.e., PP2 should *not* inhibit IL-6 protein production in 4E-BPΔ BMMØ). Since the analysis reveals the opposite, that PP2 still inhibits mAb-iB^TLR2^-induced IL-6 production in 4E-BPΔ BMMØ, FcγR signaling appears *not* to be working via inactivation of 4E-BP. Thus, further study is needed to determine the precise molecular link between FcγR ITAM signaling and IL-6 mRNA ribosome binding and translation.

**Table 1:** mAb-iB^TLR2^-induced IL-6 production in Src signaling nhibited 4E-BP knockout BMMØ.

BMMØ	mAb-iB^TLR2^	mAb-iB^TLR2^ + PP2	Inhibition by PP2
Wild Type	1,244 pg/ml ±254.3^1^	254.8 pg/ml ±27.6	79.5%
4E-BPΔ^2^	1,395 pg/ml ±10.7	222.5 pg/ml ±4.9	84.1%

1. Mean level of secreted IL-6 protein ± 1 S.D., n = 3

2. BMMØ from 4E-BP1 / 4E-BP2 / 4E-BP3 triple knockout mice

From a more global perspective, we now know that FcγR signaling can control MØ production of multiple TLR2-induced cytokines in response to recognition of mAb-iB^TLR2^ using multiple distinct mechanisms. For TNF, FcγR signaling is necessary for induction of mRNA expression (Fig 4), likely via previously discussed mechanisms. In this report, we have shown that FcγR signaling also controls cytokine (i.e., IL-6) mRNA translation. Finally, the Harton lab has recently shown that FcγR signaling can regulate the MØ IL-1β response to mAb-iB^TLR2^ by controlling inflammasome activation (which mediates conversion of pro-IL-1β to mature IL-1β) [7] consistent with prior reports on FcγR activation of IL-1β release via activation of capsase-1 activity [35, 42]. Hence, FcγR appear to play a central gubernatorial role over TLR-induced MØ production of multiple pro-inflammatory cytokines.
